# Same Factor, Different Branches: Context-Dependent Control of Dendritic Development by Lhx2

**DOI:** 10.1523/ENEURO.0296-26.2026

**Published:** 2026-10-08

**Authors:** Lauren Campbell, Ana Dorrego-Rivas

**Affiliations:** Centre for Developmental Neurobiology, Institute of Psychiatry, Psychology and Neuroscience, King’s College London, London SE1 1UL, United Kingdom

## Research highlight

The neocortex is a heterogeneous brain region, organized into anatomically and functionally specialized areas that nevertheless share a broadly conserved laminar organization and repertoire of neuronal cell classes. Morphological differences between pyramidal neurons across cortical areas have been identified in several mammalian species, while a growing body of transcriptomic evidence indicates that even excitatory neurons occupying corresponding cortical layers can differ between areas. Layer 2/3 pyramidal neurons (L2/3 PyNs) provide a useful model to examine this areal diversification: they belong to the same broad neuronal class, are located in the same cortical layer, and are generated at comparable developmental stages yet mature in distinct cortical environments. As major intracortical projection neurons, many also contribute to callosal connectivity and the exchange of information between the cerebral hemispheres. Building on these observations, Srivastava et al. (2026) asked whether L2/3 PyNs in S1 and V1 exhibit distinct morphological and physiological properties and whether the transcription factor Lhx2 contributes to their area-specific dendritic architecture.

To answer this question, the authors first compared the morphological and electrophysiological properties of S1 and V1 L2/3 PyNs in adult mice. They combined whole-cell patch-clamp recordings with biocytin-based morphological reconstruction in ex vivo mouse brain slices. S1 L2/3 PyNs had significantly longer apical dendrites than their V1 counterparts, while basal dendritic length showed a similar but nonsignificant trend. Sholl analysis further revealed an overall greater dendritic branching complexity in S1 neurons. These structural differences were accompanied by distinct intrinsic membrane and action potential properties: V1 PyNs had lower membrane capacitance and higher input resistance, consistent with their smaller dendritic arbors. They also displayed a lower rheobase and fired more action potentials in response to equivalent current injections, indicating higher intrinsic excitability than S1 PyNs. Together, these findings show that adult S1 and V1 L2/3 PyNs differ in both dendritic architecture and intrinsic electrophysiological properties despite occupying the same cortical layer.

Having identified these differences in adults, the authors next asked whether these were already present earlier in development. Using the same approach in juvenile mice, the authors found that S1 L2/3 PyNs had longer apical and basal dendrites than their V1 counterparts, while Sholl analysis revealed greater branching complexity in both compartments. V1 neurons also had higher input resistance and fired more action potentials in response to current injections, reflecting greater gain and indicating that their increased intrinsic excitability was already apparent at this stage. These results establish that the morphological and electrophysiological differences between S1 and V1 L2/3 PyNs are already present at the juvenile stage and broadly resemble those observed in adulthood.

Because dendrites receive synaptic inputs, the authors next asked whether the morphological differences between S1 and V1 L2/3 PyNs were accompanied by differences in the excitatory inputs they received. Recordings of spontaneous excitatory postsynaptic currents (sEPSCs) in juvenile ex vivo slices revealed a trend toward a higher event frequency in V1 neurons. This trend may reflect a greater density of synapses along their comparatively shorter dendritic arbors, although synapse density was not measured directly. V1 sEPSCs also showed trends toward faster rise and decay kinetics and significantly reduced charge transfer. Together, this pattern raises the possibility that excitatory inputs are organized and integrated differently by S1 and V1 L2/3 PyNs, but direct measurements of synaptic connectivity are needed to test this possibility.

Finally, given the known regulatory role of the transcription factor Lhx2 in dendritic morphogenesis (Wang et al., 2017; Bose et al., 2025), Srivastava and colleagues explored whether differences in *Lhx2* expression could contribute to the distinct dendritic architectures of S1 and V1 L2/3 PyNs. Immunostaining of E15.5 cortical sections revealed significantly higher Lhx2 protein levels in S1 progenitors than in V1 progenitors, suggesting that area-specific differences in Lhx2 levels contribute to their distinct dendritic architectures. In their previous study, the authors used in utero electroporation at E15.5 to delete *Lhx2* from S1 progenitors, markedly reducing the dendritic length and complexity of the resulting L2/3 PyNs in juvenile mice (Bose et al., 2025). Here, direct comparison showed that these *Lhx2*-deficient S1 neurons had dendritic morphologies broadly resembling those of wild-type V1 neurons. This resemblance led the authors to ask whether deleting *Lhx2* from V1 progenitors would reduce dendritic growth even further. Using the same electroporation approach, they found, surprisingly, that conditional deletion of *Lhx2* did not alter the dendritic architecture of the resulting V1 L2/3 PyNs in juvenile mice. Given the lower endogenous Lhx2 levels in V1 progenitors, the authors next tested whether increasing *Lhx2* expression could instead promote dendritic growth. *Lhx2* overexpression increased basal dendritic length and proximal branching, while apical dendrite morphology remained unchanged. These findings suggest that the influence of Lhx2 on dendritic development may depend on its expression level and/or cellular context rather than simply on its presence or absence. It would therefore be interesting to determine whether overexpressing *Lhx2* in S1 progenitors similarly enhances dendritic growth or whether endogenous Lhx2 levels there already meet or exceed the relevant functional threshold.

In conclusion, this study shows that S1 and V1 L2/3 PyNs differ in dendritic architecture and intrinsic excitability from juvenile stages into adulthood while identifying Lhx2 as a context-dependent regulator of dendritic development (Fig. 1). More intriguing than these differences is that the same transcription factor appears to have different effects in each population. Loss of *Lhx2* markedly reduces dendritic growth in S1 neurons but leaves V1 dendritic architecture unchanged, whereas increasing *Lhx2* expression in V1 selectively enhances basal dendritic growth. This asymmetry is difficult to reconcile with a simple, linear relationship between Lhx2 abundance and dendritic complexity. Instead, the effects of Lhx2 may depend on how it is interpreted within each neuronal context, through differences in transcriptional partners or accessible target genes. Direct comparisons of the transcriptional programs engaged by Lhx2 in S1 and V1 progenitors will help distinguishing these possibilities. More broadly, considered alongside transcriptomic evidence for areal diversification among cortical excitatory neurons (Tasic et al., 2018), the study suggests that neuronal diversity may arise not only from different transcription factors but also when the same factor functions differently across cortical areas.

**Figure 1.**
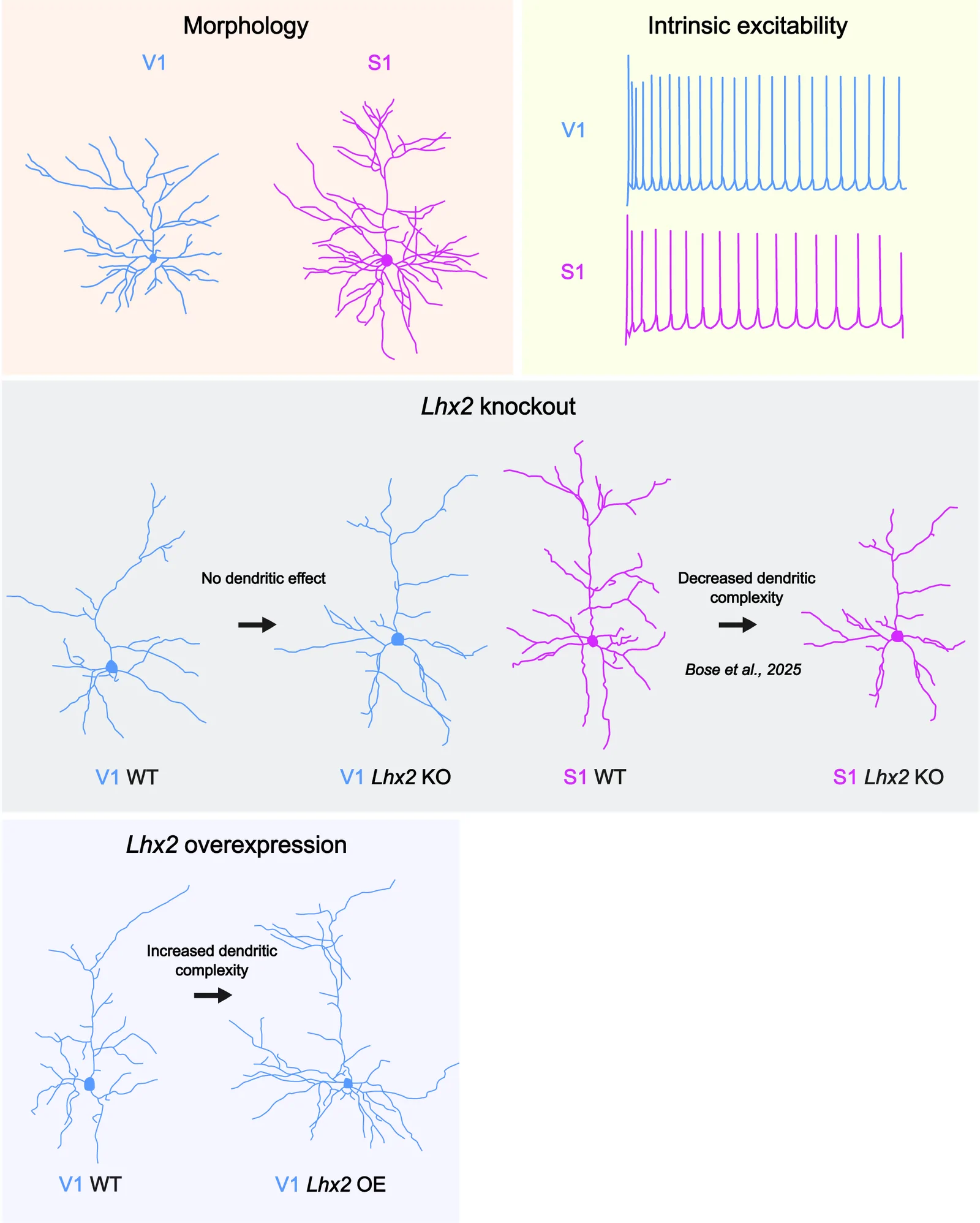
Graphical summary of the morphological and electrophysiological properties of S1 and V1 L2/3 pyramidal neurons, together with the effects of *Lhx2* deletion and overexpression.
